# Detection of Majorana fermions by Fano resonance in hybrid nanostructures

**DOI:** 10.1186/s11671-015-0914-3

**Published:** 2015-05-19

**Authors:** Jun-Jie Xia, Su-Qing Duan, Wei Zhang

**Affiliations:** Institute of Applied Physics and Computational Mathematics, Beijing, P. O. Box 8009(28), 100088 China

**Keywords:** Majorana bound states, Fano effect, Hybrid nanostructures

## Abstract

The realization and detection of Majorana fermions in condensed matter systems are of considerable importance and interest. We propose a scheme to detect the Majorana fermions by Fano resonance in hybrid nanostructures made of semiconductor quantum dots and quantum wire in proximity to superconductor. Through detailed theoretical studies of the transport properties of our hybrid nanostructures based on the non-equilibrium Green’s function technique and the equation of motion approach, it is found that the Fano resonance in the current response due to the interference among different transmission paths may give clear signature of the existence of Majorana modes. Moreover, we have found a peculiar relationship between the Fano factor *q* and the Majorana bound state coupling strength/the length of nanowire, which can be used for a design of an electronic nanoruler. Our method of detection of Majorana fermions based on Fano resonance is related to the global conductance profile, thus is robust to perturbations.

## Background

Majorana fermions (MFs) are particles which are their own antiparticles and obey non-Abelian statistics [1]. Majorana bound states (MBSs) may show inherently nonlocal nature and lead to a nonlocal electron transfer process. In recent years, MFs have attracted considerable attention due to their fundamental interest and potential applications in topological quantum computation. MFs can emerge as quasi-particle excitations in condensed matter physics [2,3]. A series of proposals have been proposed to generate the MFs, including vortex core based on fractional quantum Hall states [4-6], chiral p-wave superconductor [7,8] and superfluid [9], ultracold fermionic atoms with spin-orbit interactions [10], surfaces of three-dimensional topological insulators with proximity-induced superconductivity [11], and helical edge modes of two-dimensional topological insulators in proximity to both a superconductor and a ferromagnet [12]. One of the promising proposals is the MBSs appearing as zero-energy end states in a spin-orbit coupled one-dimensional (1D) nanowire with Zeeman spin splitting, which is in proximity to an s-wave superconductor [13-16].

Various designs have been suggested to detect and verify the existence of MBSs [17-36], for example, the thermolectric measurement [19], the conductance spectroscopy measurements [20,21], shot noise measurements [25], and nonlinear optomechanical detection [33]. In particular, the very recent observation of a zero-bias peak in the differential conductance through a semiconductor nanowire in contact with a superconducting electrode indicated the possible existence of a midgap Majorana state [21]. This zero-bias peak was also observed in subsequent experiments [26,27]. Some groups demonstrated that MBSs can be detected by coupling them to quantum dots in closed circuit. For example, the MBSs influence the conductance through the QD by inducing the sharp decrease of the conductance by a factor of 1/2, as reported by Liu and Baranger [28]. Crossed Andreev reflection (CAR) has been investigated in the double quantum dot structures, which was assisted by MBSs [29]. Seridonio et al. discussed the influence of Fano interference on the Majorana hallmark [34,35].

In spite of various theoretical and experimental studies of the generation and probe of MFs based on nanostructures, a solid clear evidence is still lacking, partially because of the similar signatures due to other effects, such as the Kondo effect [37]. In our present paper, we propose a scheme to detect the Majorana modes in hybrid nanostructures of parallel quantum dots connected by a semiconductor nanowire in proximity to a superconductor. Unlike the previous works [29-31,38-40], we focus on the Fano effect [41-49] in the QD nanowire/superconductor QD (QD-NW-QD) junction.

The tunability of several parameters of our nanostructures provides us more opportunities to explore the physics related to MFs and Fano effects. In particular, we have revealed a connection between the Fano factor and the length of nanowires, which may be used to design an electronic nanoruler. Our method of detecting Majorana modes by the Fano effect is based on the global profile of current response, thus is robust to external perturbations.

## Methods

### The model and theoretical formalism

**Figure 1:**
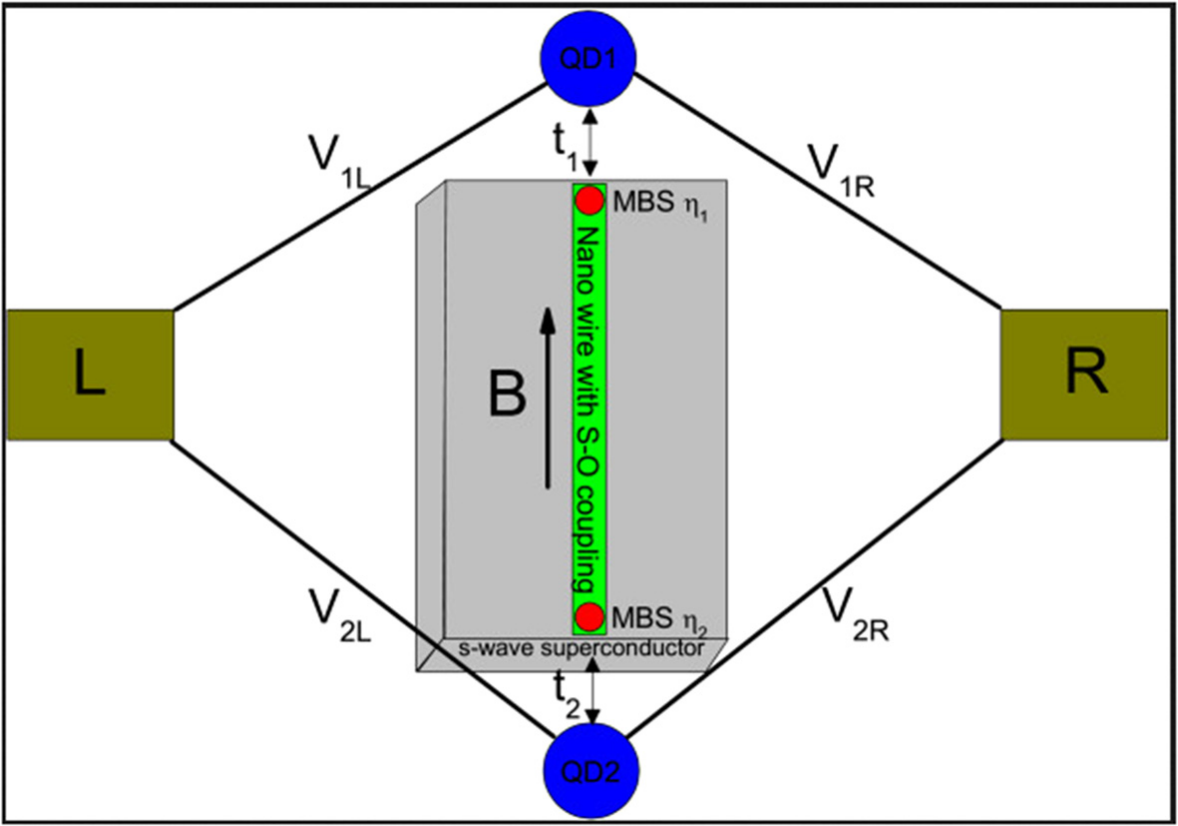
Schematic diagram of the QD-NW-QD system. The MBSs locate at the two ends of the nanowire with modest Zeeman splitting and strong spin-orbit coupling, which is in proximity to an s-wave superconductor.

As schematically shown in Figure 1, the hybrid system consists of two quantum dots with spinless electronic states coupled by a semiconductor nanowire with strong Rashba spin-orbit interaction, a modest magnetic field *B*, and in proximity to an s-wave superconductor. The MBSs as electron-hole quasiparticle excitations can appear at the two ends of such a nanowire. When the Zeeman splitting energy *V*_*z*_=*gμ*_*B*_*B*, the proximity-induced order parameter *Δ*, and the chemical potential *μ* satisfy the condition $V_{z}>\sqrt {\Delta ^{2}+\mu ^{2}}$, the nanowire is driven into the topological superconducting phase, and a pair of zero-energy MBSs would emerge at each end of the nanowire [15]. Then, the two quantum dots which are tunnel-coupled to the ends of the nanowire are now effectively coupled to the MBSs. The total Hamiltonian of the QD-NW-QD system can be written as:

(1) $$ H=H_{\text{system}}+H_{\text{leads}}+H_{T}.  $$

Here, the first term *H*_system_ describes the tunneling-coupled MBSs and quantum dots:

(2) $$\begin{array}{@{}rcl@{}} H_{\text{system}}&=&\sum\limits_{j=1,2}\varepsilon_{j}d_{j}^{\dagger}d_{j}+\frac{i}{2}\epsilon_{M}\eta_{1}\eta_{2}+ t_{1}(d_{1}-d_{1}^{\dagger})\eta_{1}\\&&+\,it_{2}(d_{2}+d_{2}^{\dagger})\eta_{2}, \end{array} $$

where $d_{j}^{\dagger }(d_{j})$ is the electron creation (annihilation) operator of the *j*th quantum dot, *η*_1_ and *η*_2_ are the Majorana operators, and the parameter $\epsilon _{M}\propto e^{-2l/\xi _{0}}\cos (k_{F}l)$ describes the coupling energy between the two MBSs [50], with *k*_F_ the Fermi wave vector, *ξ*_0_ the superconducting coherence length, *l* the nanowire length, and *t*_1_(*t*_2_) represents the coupling strength between the first (second) QD and the MBS *η*_1_(*η*_2_). For the sake of calculation convenience, one can transform the Majorana operator to the regular fermion representation, using the relations *η*_1_=*f*+*f*^*†*^ and *η*_2_=*i*(*f*^*†*^−*f*). In this regular fermion representation, the system Hamiltonian is accordingly rewritten as:

$$\begin{array}{@{}rcl@{}} H_{\text{system}}&=&\sum\limits_{j=1,2}\varepsilon_{j}d_{j}^{\dagger}d_{j}+\epsilon_{M}(f^{\dagger}f-\frac{1}{2}) \\&&+\left[t_{1}(d_{1}-d_{1}^{\dagger})f+t_{2}(d_{2}+d_{2}^{\dagger})f+H.c.\right]. \end{array} $$

The Hamiltonian for the two electrodes is:

(3) $$\begin{array}{@{}rcl@{}} H_{\text{leads}}=\sum\limits_{\alpha=L,R}\sum\limits_{k}\varepsilon_{\alpha k}c_{\alpha k}^{\dagger}c_{\alpha k}, \end{array} $$

where $ c_{\alpha k}(c_{\alpha k}^{\dagger }) $ is the annihilation (creation) operator for the electron in the *α* lead. The term *H*_*T*_ accounts for the tunneling between the dots and the leads:

(4) $$\begin{array}{@{}rcl@{}} H_{T}=\sum\limits_{\alpha=L,R}\sum\limits_{j=1,2}V_{\textit{j}\alpha}d_{j}^{\dagger}c_{\alpha k}+H.c., \end{array} $$

with *V*_*jα*_ the coupling strength between the *j*th dot and the *α* electrode. We investigate the transport properties of the QD-NW-QD system in the presence of a bias voltage *V*_*b*_ between the two leads with *μ*_*L*_=*ε*_F_+*eV*_*b*_ and *μ*_*R*_=*ε*_F_ (*ε*_F_ is the Fermi level which is assumed to be zero). With the help of the equation of motion method, Green’s function of the system can be calculated in the Nambu representation [28,38]:

(5) $$\begin{array}{@{}rcl@{}} G^{r}\left(\omega\right)=\frac{1}{\omega-H_{\text{system}}-\Sigma_{\text{leads}}^{r}}, \end{array} $$

where $\Sigma _{\text {leads}}^{r}=-\frac {i}{2}\sum _{\alpha }\left (\Gamma _{e}^{\alpha }+\Gamma _{h}^{\alpha }\right)$ is the self-energy due to the leads. And $\Gamma _{e}^{\alpha }(\Gamma _{h}^{\alpha })$ are 6×6 matrices describing the coupling of particle(hole) to *α* lead:

$$\begin{array}{@{}rcl@{}} \Gamma_{e,mn}^{\alpha} &=&\Gamma_{\alpha 1}\delta_{1m}\delta_{1n}+\Gamma_{\alpha 2}\delta_{5m}\delta_{5n}+\sqrt{\Gamma_{\alpha 1}\Gamma_{\alpha 2}}(\delta_{1m}\delta_{5n}\\&&+\delta_{5m}\delta_{1n}) \\ \Gamma_{h,mn}^{\alpha} &=&\Gamma_{\alpha 1}\delta_{2m}\delta_{2n}+\Gamma_{\alpha 2}\delta_{6m}\delta_{6n}+\sqrt{\Gamma_{\alpha 1}\Gamma_{\alpha 2}}(\delta_{2m}\delta_{6n}\\&&+\delta_{6m}\delta_{2n}), \end{array} $$

where *Γ*_*αj*_≡2*π*|*V*_*jα*_|^2^*ρ*_*α*_ is the dot-lead coupling and *ρ*_*α*_ is the density of states of the *α* lead. Once Green’s function is obtained, the current from the left lead can be calculated:

(6) $$\begin{array}{@{}rcl@{}} I_{L}&=&\frac{e}{h}\int d\omega\left[T_{ee}^{LR} \left(\omega\right) \left({f_{e}^{L}}-{f_{e}^{R}} \right) + T_{eh}^{LL} \left(\omega\right) \left({f_{e}^{L}}-{f_{h}^{L}}\right)\right. \\ &&\left.+T_{eh}^{LR}\left(\omega\right)\left({f_{e}^{L}}-{f_{h}^{R}}\right)\right]. \end{array} $$

In this formula, $f_{e}^{\alpha }$ and $f_{h}^{\alpha }$ are the Fermi distribution functions of the electron and hole in *α* lead and *G*^*a*^=*G*^*r*^^*†*^. $T_{\textit {ee}}^{LR}\left (\omega \right)=Tr[G^{r}{\Gamma _{e}^{R}}G^{a}{\Gamma _{e}^{L}}]$ is the transmission coefficient which is contributed by the electron teleportation process from the left lead to the right lead, while $T_{\textit {eh}}^{LL}\left (\omega \right)=Tr[G^{r}{\Gamma _{h}^{L}}G^{a}{\Gamma _{e}^{L}}]$ is the transmission coefficient in the left lead arising from the local Andreev reflection (AR), and $T_{\textit {eh}}^{LR}\left (\omega \right)=Tr[G^{r}{\Gamma _{h}^{R}}G^{a}{\Gamma _{e}^{L}}]$ is the transmission coefficient due to the contribution of CAR. Some additional details of the theoretical formulism are included in Appendix 1.

## Results and discussion

### Tunable Fano effect

With the formulation developed in the above section, analytical/numerical calculation has been performed to investigate the zero-temperature transport properties of the QD-NW-QD system. For simplicity, in this paper, we mainly consider the symmetric configurations, i.e., *ε*_1_=*ε*_2_=*ε*_0_, *Γ*_*α*1_=*Γ*_*α*2_=*Γ*(*α*=*L*,*R*), *t*_1_=*t*_2_=*t*. *t* is set as the unit of energy.

#### Case I without interaction between MFs

**Figure 2:**
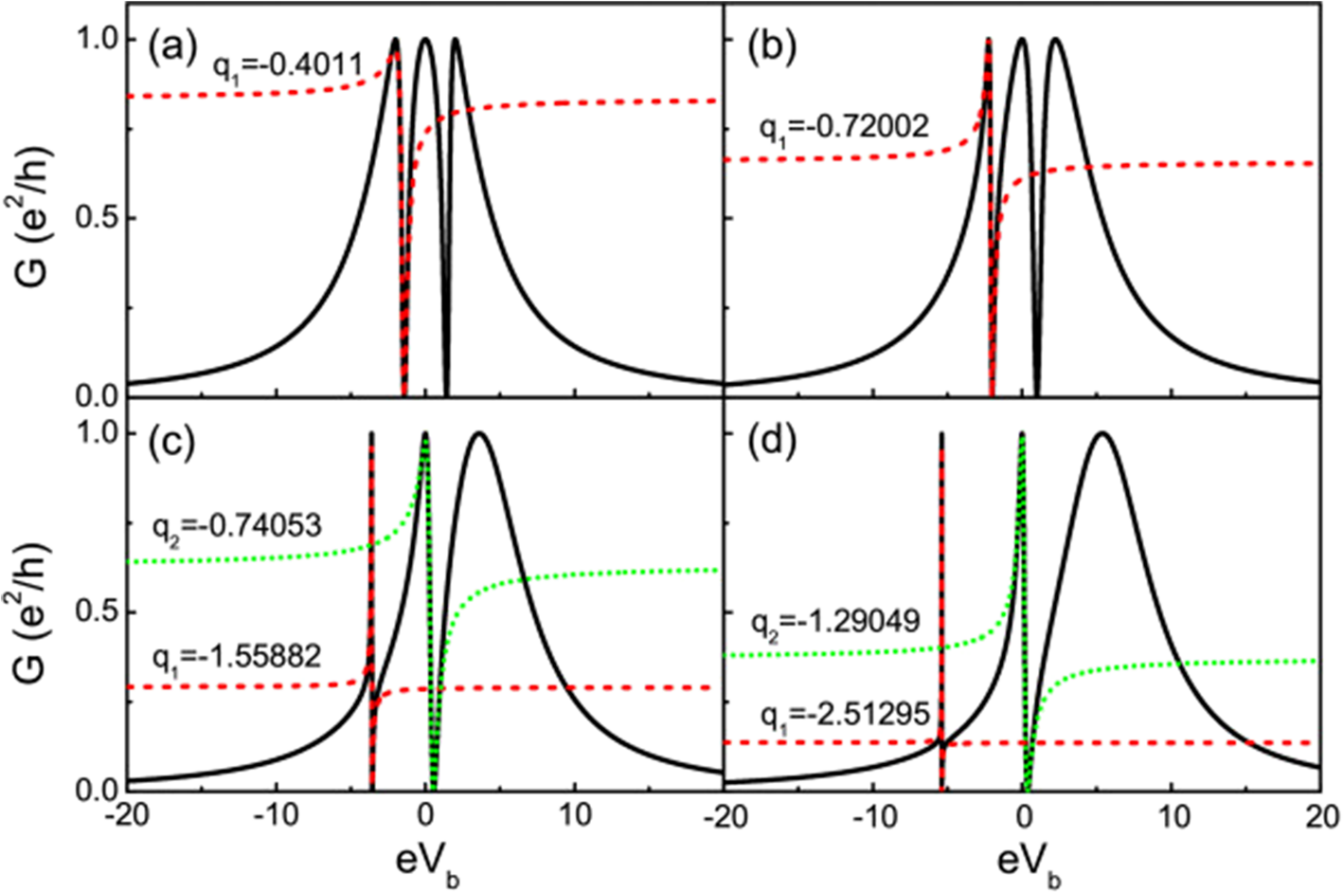
Conductance curve of the QD-NW-QD system with different electron energy levels in the quantum dots. The dashed lines are the fitting Fano lineshapes. **(a)** *ε*_0_=0, **(b)** *ε*_0_=1, **(c)** *ε*_0_=3, and **(d)** *ε*_0_=5. The coupling of the MBSs is *ε*_*M*_=0 and the dot-lead coupling *Γ*=2.0.

First, the case of a long nanowire with *ε*_*M*_=0 is considered. In this situation, the conductance from AR (consisted of local AR and CAR) is completely suppressed, which can be proved by some algebra. The electron teleportation conductance takes the form:

(7) $$  G=\frac{e^{2}}{h}\frac{4\Gamma^{2}[2t^{2}-\omega(\varepsilon_{0}+\omega)]^{2}}{4\Gamma^{2}[2t^{2}-\omega(\varepsilon_{0}+\omega)]^{2}+\omega^{2}(\Delta^{2}-\omega^{2})^{2}} \Big|_{\omega=eV_{b}},  $$

where $\Delta =\sqrt {{\varepsilon _{0}^{2}}+4t^{2}}$. Figure 2 shows the conductance for various electron energy levels in quantum dots. One can see that all the conductance curves exhibit three peaks at the positions of the effective molecular states (with energies $\omega =\pm \sqrt {{\varepsilon _{0}^{2}}+4t^{2}}$ and *ω*=0) of the QD-NW-QD system, which provide three special transmission paths for the transport. For details about the molecular states, see Appendix 2. In Figure 2, the conductance lineshape varies with the energy level of the quantum dots exotically, which may be tuned by gate voltage in experiment. This phenomenon is quite different with the case of directly parallel coupled double quantum dot system without the Majorana fermions [51,52]. The conductance curve of the directly parallel coupled double quantum dot shifts trivially with the energy level of QDs (see Appendix 3). The conductance of our system exhibits typical Breit-Wigner and Fano resonances. In Figure 2a, the two antisymmetric peaks locate at the positions of *eV*_*b*_=±*Δ*. However, the antisymmetric peaks locate at the positions of *eV*_*b*_=−*Δ*, *eV*_*b*_=0 in Figure 2b,c,d. One may obtain the Fano factors (*q*_1_ for Fano resonance at *eV*_*b*_=−*Δ* in Figure 2a,b,c,d and *q*_2_ for Fano resonance at *eV*_*b*_=0 in Figure 2c,d) by fitting to the Fano function. The absolute value of the Fano factor *q*_1_ increases monotonically with increasing the energy level of QDs.

**Figure 3:**
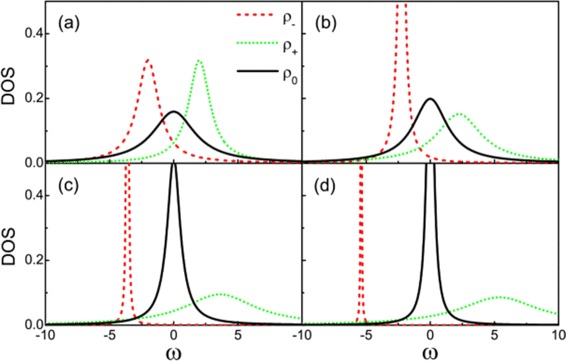
The density of states for three molecular states.**(a)** *ε*_0_=0, **(b)** *ε*_0_=1, **(c)** *ε*_0_=3, and **(d)** *ε*_0_=5. Other parameters are the same with those of Figure 2.

Additional insight into the physics underlying these results can be obtained by examining the density of states (DOSs) of the effective molecular states. The algebra of the DOS is not particularly enlightening so only numerical results are presented here. One can find the analytic details in Appendix 2. Figure 3 displays how the DOSs of the effective molecular states change with the electron energy level of the quantum dots. For the state at *ω*=0, it is only composed of Majorana modes and the other two states consist of Dirac modes. When *ε*_0_=0, the two MBSs are spatially isolated on each of the two dots. In Figure 3a with *ε*_0_=0, the DOS of the molecular state at *ω*=0 which consists of two Majorana states is wider than the DOSs of the other two states at *ω*=±*Δ*. The interference between the wide Majorana molecular state channel and the other narrow molecular state channels leads to the Fano resonance. Figure 3 shows that the widths of DOSs of the molecular states at *ω*=−*Δ* and *ω*=0 decrease with the increase of the QD energy level *ε*_0_. And the width of DOS of the molecular state at *ω*=*Δ* increases with the increase of the QD energy level *ε*_0_. When $\varepsilon _{0}=\frac {t}{\sqrt {2}}$, the two molecular states at *ω*=*Δ* and *ω*=0 have the equal width (This situation is not shown in Figure 3). If the energy level of QDs is tuned above $\frac {t}{\sqrt {2}}$, the molecular state at *ω*=*Δ* would be the widest transport path. So the conductance curve will display two antisymmetric peaks locating at *eV*_*b*_=−*Δ*, *eV*_*b*_=0 in Figure 2c,d. In the region of *eV*_*b*_∼−*Δ*, one can simplify the formula of conductance, i.e.,

(8) $$\begin{array}{@{}rcl@{}} G\approx A_{1}\frac{(\frac{\omega+B_{1}}{C_{1}}+{q}_{1})^{2}}{(\frac{\omega+B_{1}}{C_{1}})^{2}+1} \Big|_{\omega=eV_{b}}, \end{array} $$

where:

$$\begin{array}{@{}rcl@{}} A_{1} &=&\frac{\Gamma^{2}(\varepsilon_{0}-2\Delta)^{2}}{\Gamma^{2}(\varepsilon_{0}-2\Delta)^{2}+\Delta^{4}}, \\B_{1} &=&\Delta+\frac{1}{2}\frac{\Gamma^{2}(\varepsilon_{0}-2\Delta)(\varepsilon_{0}-\Delta)^{2}}{\Gamma^{2}(\varepsilon_{0}-2\Delta)^{2}+\Delta^{4}}, \\ C_{1} &=&\frac{\Gamma}{2}\frac{(\varepsilon_{0}-\Delta)^{2}\Delta^{2}}{\Gamma^{2}(\varepsilon_{0}-2\Delta)^{2}+\Delta^{4}}, \,\quad\quad{q}_{1} =\frac{\Delta^{2}}{\Gamma(\varepsilon_{0}-2\Delta)}. \end{array} $$

From the above equations, it is clear that the conductance has standard Fano lineshape, which describes well the conductance near the resonance. The analytical Fano factor agrees well with that obtained by numerical fitting, and the absolute value of the Fano factor *q*_1_ increases with the increase of electron energy level in quantum dots. In the case of *V*_*b*_→0, the conductance formula can be simplified as:

(9) $$\begin{array}{@{}rcl@{}} G\approx A_{2}\frac{(\frac{\omega+B_{2}}{C_{2}}+{q}_{2})^{2}}{(\frac{\omega+B_{2}}{C_{2}})^{2}+1} \Big|_{\omega=eV_{b}}, \end{array} $$

where:

$$\begin{array}{@{}rcl@{}} A_{2} &=&\frac{4\Gamma^{2}{\varepsilon_{0}^{2}}}{4\Gamma^{2}{\varepsilon_{0}^{2}}+\Delta^{4}}, \qquad\quad\quad B_{2} =\frac{-8\Gamma^{2}t^{2}\varepsilon_{0}}{4\Gamma^{2}{\varepsilon_{0}^{2}}+\Delta^{4}}, \\ C_{2} &=&\frac{4\Gamma t^{2}\Delta^{2}}{4\Gamma^{2}{\varepsilon_{0}^{2}}+\Delta^{4}}, \qquad\quad\quad {q}_{2} =\frac{-\Delta^{2}}{2\Gamma\varepsilon_{0}}. \end{array} $$

Equations (9) and (10) are valid in the range of *ε*_0_≫*t*. In this regime, *q*_1_∼2*q*_2_. Numerical results in Figure 2c,d also show the same relation. One may note that the antisymmetric lineshape of the zero bias peak (i.e., the antisymmetric peak at *V*_*b*_=0) could be used to detect the existence of the Majorana modes.

#### Case II with interaction between MFs

**Figure 4:**
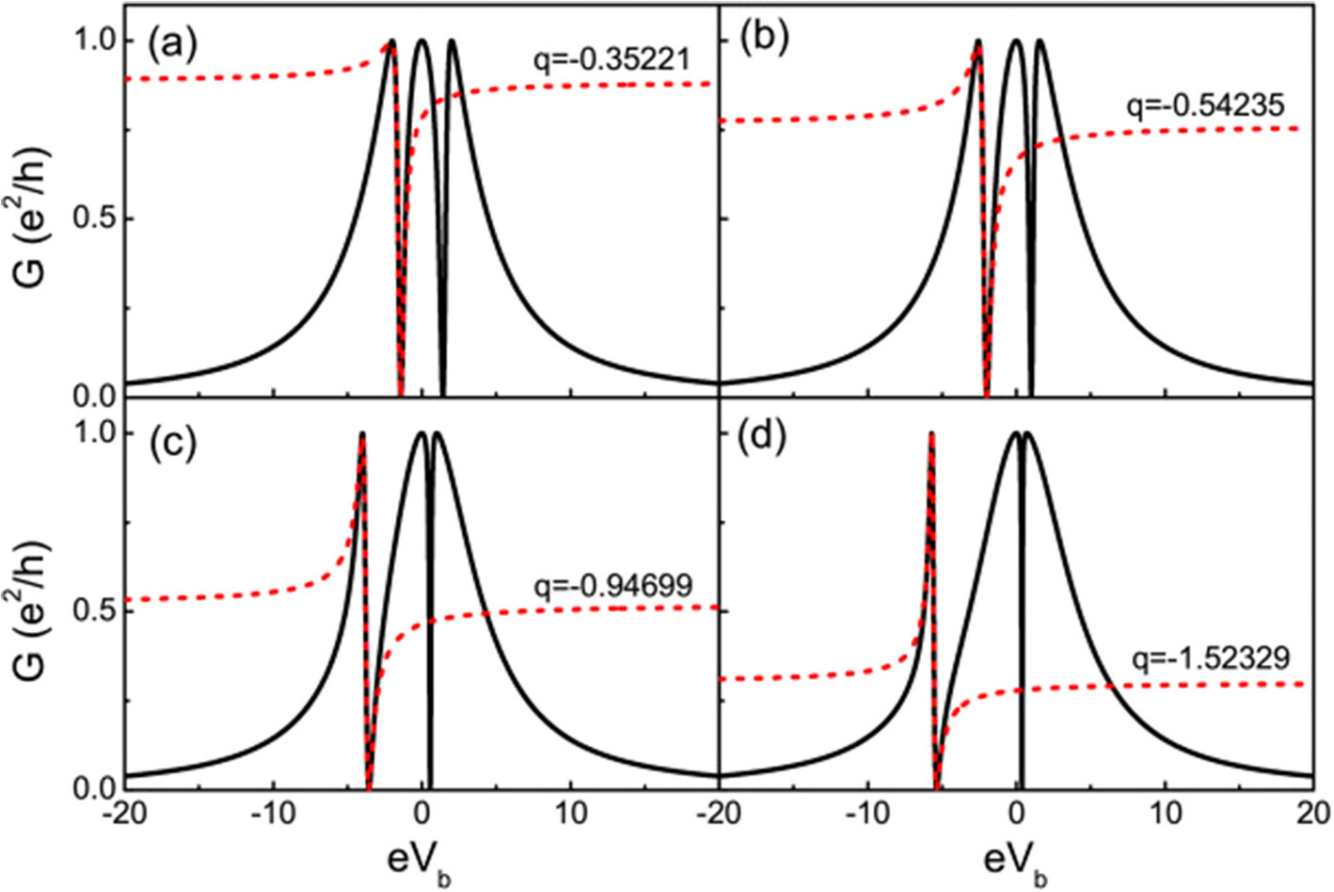
Conductance curve of the QD-NW-QD system with different MBS coupling strength. The dashed lines are the fitting Fano lineshapes. **(a)** *ε*_*M*_=0, **(b)** *ε*_*M*_=1, **(c)** *ε*_*M*_=3, and **(d)** *ε*_*M*_=5. Other parameters: *ε*_0_=0 and *Γ*=2.0.

If the nanowire is not long enough, the two MBSs living in the two ends of the wire couple to each other. Figure 4 depicts the conductance spectra with nonzero coupling between the two MBSs. Here, the energy levels of the two QDs are tuned align with the Fermi energy (i.e., *ε*_0_=0). Also, the conductance coming from electron teleportation survives, and the other two processes are suppressed. The conductance takes the form:

(10) $$ {\fontsize{8.8}{6}\begin{aligned} G=\frac{e^{2}}{h}\frac{\Gamma^{2}[4t^{2}-2\epsilon_{M}\omega-\omega^{2}]^{2}}{\Gamma^{2}[4t^{2}-2\epsilon_{M}\omega-\omega^{2}]^{2} +\omega^{2}(4t^{2}-\epsilon_{M}\omega-\omega^{2})^{2}} \Big|_{\omega=eV_{b}}. \end{aligned}}  $$

The conductance curve exhibits three peaks, which are three molecular states locating at *eV*_*b*_=0 and $eV_{b}=\frac {\pm \kappa -\epsilon _{M}}{2}$, where $\kappa =\sqrt {{\epsilon _{M}^{2}}+16t^{2}}$. Detailed description about molecular states can be found in Appendix 4. In Figure 4, one can see with the increase of the *ε*_*M*_, the peaks at *eV*_*b*_=0 and $eV_{b}=\frac {\kappa -\epsilon _{M}}{2}$ go close. The conductance displays clearly Fano resonance, which comes from the interference between three molecular states. And the effective antisymmetric factor *q* can be obtained by fitting to Fano function. The absolute value of the factor *q* increases with increasing the two MBS coupling strength *ε*_*M*_, which sometimes means shortening the length of the nanowire. To explain this result, one can use the simplified formula of conductance in the region of $eV_{b} \to \frac {-\kappa -\epsilon _{M}}{2}$, i.e.,

$$\begin{array}{@{}rcl@{}} G \approx A_{3}\frac{(\frac{\omega+B_{3}}{C_{3}}+{q}_{3})^{2}}{(\frac{\omega+B_{3}}{C_{3}})^{2}+1} \Big|_{\omega=eV_{b}}, \end{array} $$

which is valid in the case of *ε*_*M*_≫*t* with:

$$\begin{array}{@{}rcl@{}} A_{3} &=&\frac{16\Gamma^{2}}{(\epsilon_{M}+\kappa)^{2}+16\Gamma^{2}}, \qquad\quad\quad \\B_{3}&=&\frac{2t^{2}}{\kappa}\frac{16\Gamma^{2}}{(\epsilon_{M}+\kappa)^{2}+16\Gamma^{2}}+\frac{\epsilon_{M}+\kappa}{2},\\ C_{3} &=&\frac{-8\Gamma t^{2}(\epsilon_{M}+\kappa)}{\kappa((\epsilon_{M}+\kappa)^{2}+16\Gamma^{2})}, \qquad\quad q_{3} =-\frac{\epsilon_{M}+\kappa}{4\Gamma}. \end{array} $$

**Figure 5:**
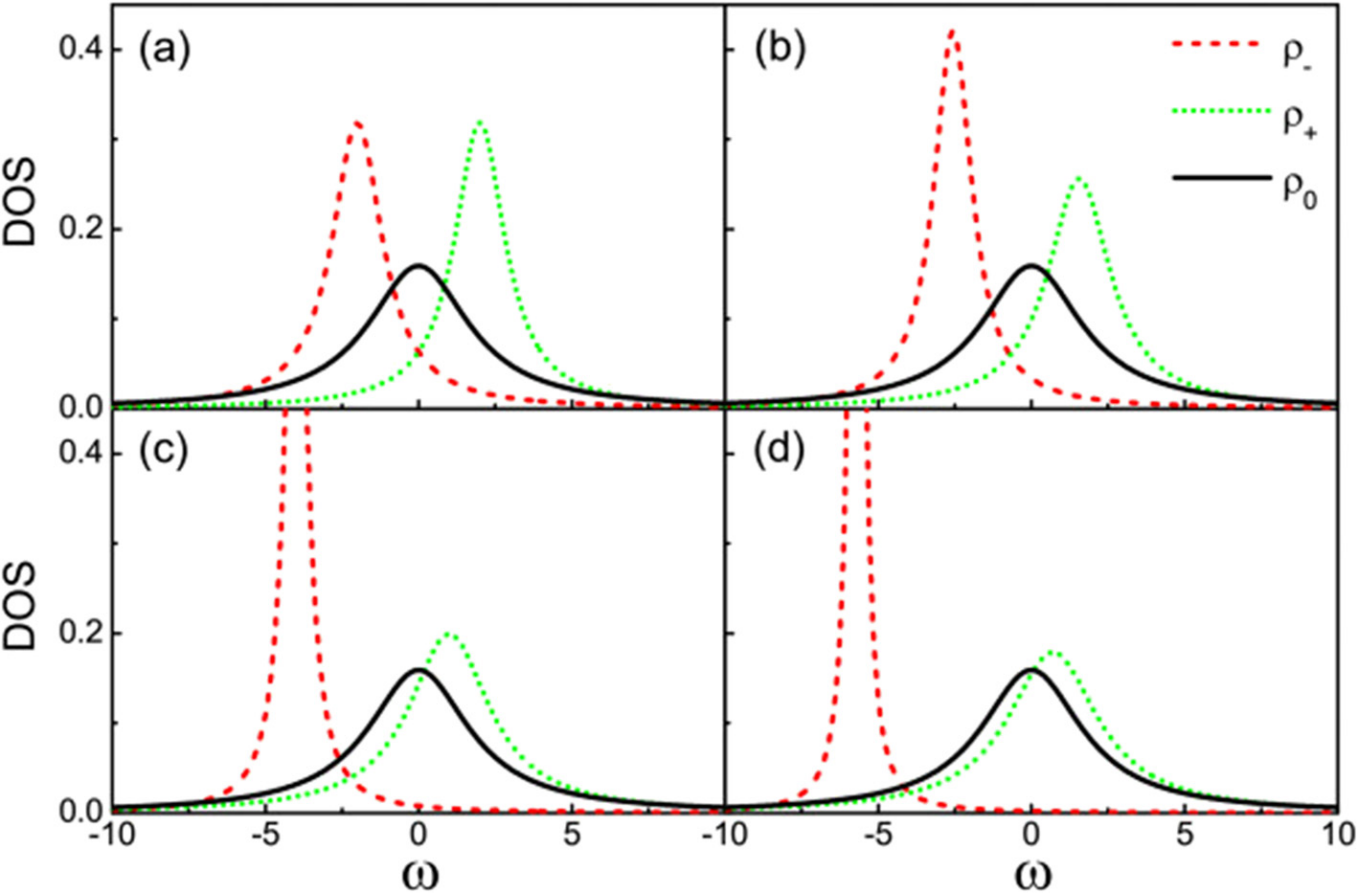
The density of states of three molecular states.**(a)** *ε*_*M*_=0, **(b)** *ε*_*M*_=1, **(c)** *ε*_*M*_=3, and **(d)** *ε*_*M*_=5. Other parameters are the same as in Figure 4.

The absolute value of the Fano factor increases with the increase of MBS coupling strength *ε*_*M*_. Figure 5 displays the DOSs of effective molecular states corresponding to the three transmission paths. One sees that the density of state at *ω*=0 (which is composed of two Majorana states) is invariant with the change of *ε*_*M*_. It is because the two Majorana modes are localized on each of the two quantum dots and do not vary with the change of *ε*_*M*_. The DOSs of the other two molecular states change monotonically with the increase of the *ε*_*M*_. The broadening of one molecular state is always accompanied with the shrinking of another molecular state. With the increase of *ε*_*M*_, the state at $\omega =\frac {\kappa -\epsilon _{M}}{2}$ nearly has the same width with the state at *ω*=0. So only one distinct Fano lineshape can be observed in the conductance spectrum, and the Fano factor can be obtained from the conductance curve. This peculiar relationship between the Fano factor and the two MBS coupling strength can be used to measure the length of the nanowire.

The coupling strength of the MBSs is determined by [50]:

(11) $$\begin{array}{@{}rcl@{}} \epsilon_{M} \approx \hbar^{2} k_{F} \frac{e^{-\frac{2L}{\xi}}} {m^{*} \xi} \cos(k_{F} L), \end{array} $$

where *k*_F_ is the Fermi wave vector and *ξ* is the superconducting coherence length:

$$\begin{array}{@{}rcl@{}} k_{\mathrm{F}}\approx\frac{2}{\alpha_{R}}\sqrt{\sqrt{(\mu_{\text{eff}}+\frac{m^{\ast}{\alpha_{R}^{2}}}{\hbar^{2}})^{2}+{V_{z}^{2}}-\Delta^{2}-\mu_{\text{eff}}^{2}}+\mu_{\text{eff}}}, \end{array} $$

$$\begin{array}{@{}rcl@{}} \xi\approx\frac{\hbar^{2}}{m^{\ast}\alpha_{R}}\frac{\sqrt{(\mu_{\text{eff}}+\frac{m^{\ast}{\alpha_{R}^{2}}}{\hbar^{2}})^{2}+{V_{z}^{2}}-\Delta^{2}-\mu_{\text{eff}}^{2}}}{\Delta}. \end{array} $$

**Figure 6:**
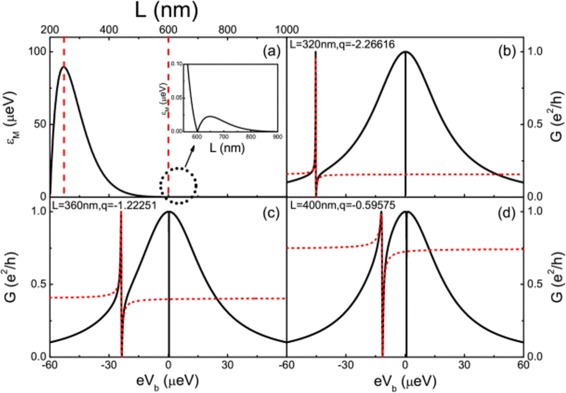
The MBS coupling strength *ε*_*M*_ and conductance curves of the realistic coupled QD-NW-QD systems.**(a)** The MBS coupling strength *ε*_*M*_ versus the length of the InSb nanowire with the parameters: the induced superconducting gap *Δ*=0.375 meV, the Zeeman field *B*=0.178 T, and the chemical potential *μ*_eff_=0.006 meV. The inset shows the length range of 550 to 900 nm. **(b-d)** Conductance curves of the realistic coupled QD-NW-QD systems with different MBS coupling strength corresponding to different lengths of the InSb nanowire. The dashed lines are the fitting Fano lineshapes. The energy levels of the two quantum dots are in line with the Fermi level *ε*_1_=*ε*_2_=0, *t*=2 µeV and *Γ*_*L*1_=*Γ*_*L*2_=*Γ*_*R*1_=*Γ*_*R*2_=10 µeV.

**Figure 7:**
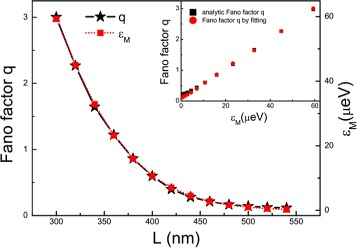
Dependence of the Fano factor *q* and MBS coupling strength on the length of the InSb nanowire. The inset depicts the Fano factor as a function of *ε*_*M*_. The parameters are the same as those in Figure 6.

Here, we consider a realistic InSb nanowire with *m*^∗^=0.015*m*_*e*_, *α*_*R*_=0.2*eV* Å, *a*≈5.3 Å, and *g*=50 [15,53]. Tuning the induced superconducting gap *Δ*=0.375 meV, the Zeeman field *B*=0.178 T, and the chemical potential *μ*_eff_=0.006 meV. The Fermi wave vector of the superconducting nanowire is *k*_F_≈0.0078 nm ^−1^, and the superconducting coherence length is *ξ*≈97 nm. The coupling strength of the the MBSs as a function of the nanowire length is shown in Figure 6a. In general, there may be other parameters, *t*_1_and *t*_2_, that depend on *L*. When the *L* is not very small (in our concerned regime (*L* larger than 250 nm), see Figures 6a and 7), the MF is well localized near the edge of the nanowire, then there is no distinct dependence of *t*_*i*_ on *L*.

Figures 6b,c,d shows the conductance curves with different lengths of the superconducting nanowire. As we have shown, different coupling strengths of the MBSs lead to different Fano factors. Figure 7 exhibits the relationship between the length of nanowire and the Fano factor *q*. If the length of the nanowire is within a certain region (the region is from 250 to 600 nm in our present case in Figure 6a), the length of the nanowire and the Fano factor *q* have simple and unique relation. In the case of *ε*_*M*_≫*t*, which means the nanowire is not too long, the Fano factor *q* and the MBS coupling strength *ε*_*M*_ have linear relationship with the slope controlled by *Γ*, dot-lead coupling strength. Then, the transport signal can be used to measure the length at nanometer scale. This method have particular advantage. Fano lineshape is a global profile, which is based on many data in a collective way. Thus, the overall lineshape is insensitive to the fluctuation of each data and it is robust to noise.

### Discussion

**Figure 8:**
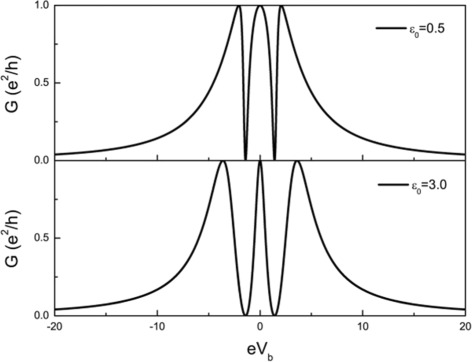
The conductance curve of the QD-NW-QD system with particle-hole symmetry, i.e., *ε*_1_=−*ε*_2_=*ε*_0_. **(a)** *ε*_0_=0.5 and **(b)** *ε*_0_=3. The coupling of the MBSs is *ε*_*M*_=0 and the dot-lead coupling *Γ*=2.0.

We have mainly considered the symmetric configurations with the same energy levels of the two quantum dots. Another interesting situation is the special antisymmetric configuration with *ε*_1_=−*ε*_2_=*ε*_0_. In the experiment, the energy levels of the quantum dots can be tuned by applying appropriate gate voltage. Interestingly, the spacial asymmetric system shows different behaviors due to the presence of particle-hole symmetry [54,55]. As one can see from Figure 8, the conductance curve exhibits three peaks, and there always exists one symmetric peak at *eV*_*b*_=0. This symmetric peak is the result of coherent interference from the three effective molecular states, which may be viewed as the superposition of two asymmetric Fano peaks. It is the particle-hole symmetry that leads to the recovery of the symmetric lineshape of the central peak. We note that the detection of MF by Fano effect with one MF coupled to a QD/adatom in an interferometer was proposed in Ref. [34,35], while two MFs couple to QDs and are involved directly in the transport in our system, which leads to new transport features (for example, the gate voltage tunable conductance lineshape, in particular, the particle-hole symmetry related recovery of symmetric lineshape due to the superposition of two asymmetric Fano peaks) due to different structures and symmetries.

**Figure 9:**
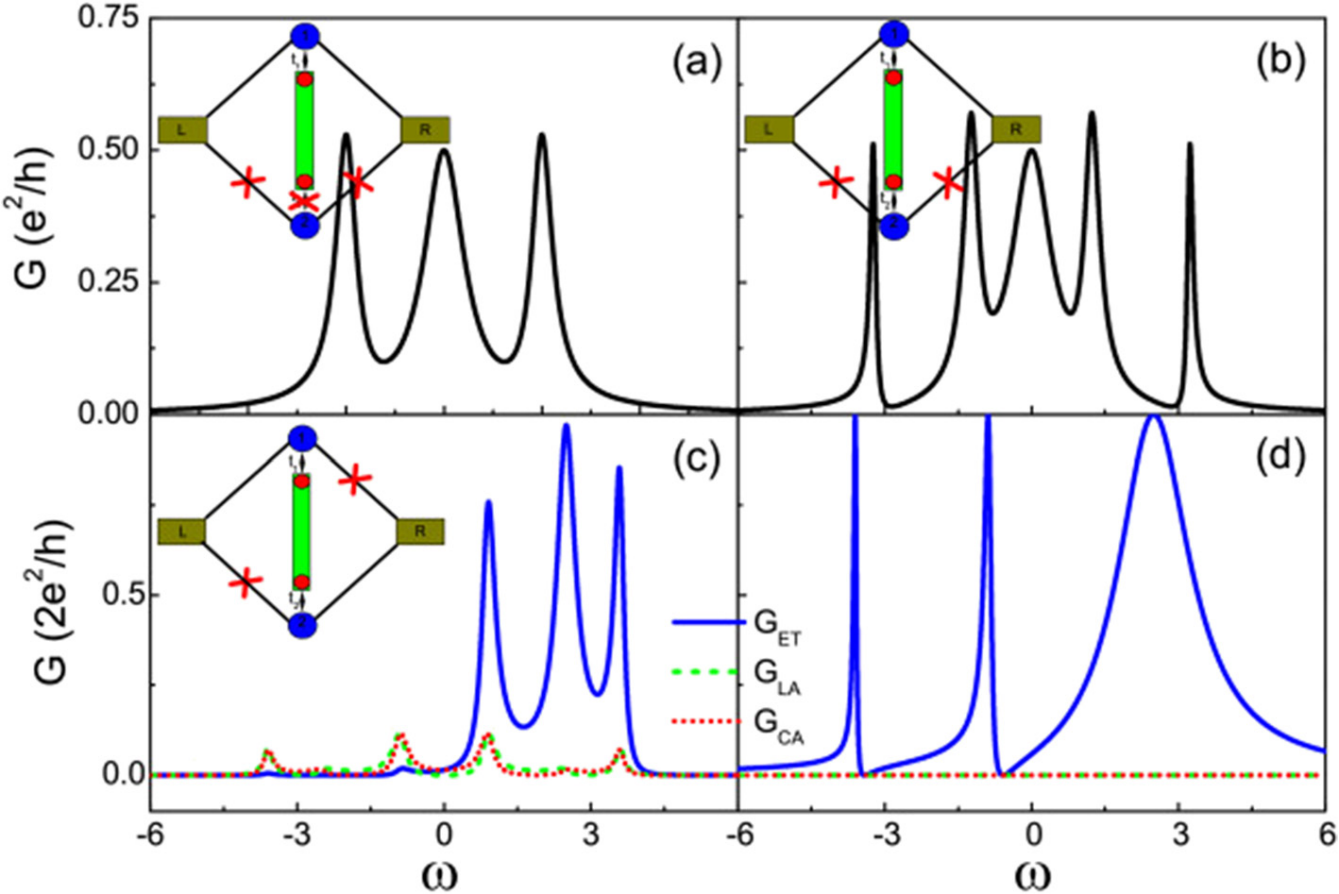
The comparison with other cases.**(a)** The conductance curve of the case *ε*_*M*_=0, *Γ*_*L*2_=*Γ*_*R*2_=*t*_2_=0. **(b)** The conductance curve of the case *Γ*_*L*2_=*Γ*_*R*2_=0. The bias voltage *V*_*b*_ between the two leads is set as $\mu _{L}=\varepsilon _{\mathrm {F}}+\frac {eV_{b}}{2}$ and $\mu _{R}=\varepsilon _{\mathrm {F}}-\frac {eV_{b}}{2}$. *t*_2_=1.0, *ε*_*M*_=2.0. Other parameters: *Γ*_*L*1_=*Γ*_*R*1_=0.5, *ε*_1_=*ε*_2_=0. **(c**, **d)** The conductance for each process: *G*_ET_ is the conductance from electron teleportation process, *G*_LA_ is the conductance from local AR process, and *G*_CA_ is the conductance from CAR process. (c) *Γ*_*L*2_=*Γ*_*R*1_=0 and (d) *Γ*_*L*2_=*Γ*_*R*1_=0.5. Other parameters: *Γ*_*L*1_=*Γ*_*R*2_=0.5, *ε*_*M*_=2.0, *ε*_1_=*ε*_2_=2.0. The coupling strength between the quantum dot and the MBS *t* is set as the unit of energy.

Our model contains much interesting physics. Several systems/models investigated in the literature are related to our model. One may set *Γ*_*L*2_=*Γ*_*R*2_=*t*_2_=0, and the case is reduced to that considered by Liu and Baranger [28]. If we set *Γ*_*L*2_=*Γ*_*R*2_=0, then the model is reduced to the case investigated by Li and Bai [56] where the second quantum dot is decoupled from the leads. As seen in Figure 9a,b, the conductance curves show the famous symmetric zero-bias peak that is reduced by a factor of 1/2. In Figure 9b, the other QD adds more modes involved in the transport, which results in two more peaks. The coupling to the additional states leads to the shift of molecular states or the peaks in the conductance curve. These results are consistent with the model discussed by Li and Bai [56]. If we set *Γ*_*L*2_=*Γ*_*R*1_=0, the transport property of N-QD-NW-QD-N junction can be realized [38] (see Figure 9c). It is seen that the local AR and CAR can be partially suppressed by tuning the parameters of the system. The contribution of local AR and CAR processes to the conductance has also been addressed in Ref. [31]. However, the local AR and CAR can be suppressed exactly in our model when *ε*_1_=*ε*_2_ (see Figure 9d). Here, one can use the molecular basis to demonstrate the *complete* suppression of AR. In the molecular basis, Green’s function is block diagonal:

$$\begin{array}{@{}rcl@{}} G^{r/a}=\left(\begin{array}{cc} G_{A}^{r/a} & 0 \\ 0 & G_{B}^{r/a} \end{array} \right). \end{array} $$

And the self-energies are also block diagonal, i.e.,

$$\begin{array}{@{}rcl@{}} \Gamma_{e}^{\alpha}=\left(\begin{array}{cc} \Gamma_{e,A}^{\alpha} & 0 \\ 0 & 0 \end{array} \right), \Gamma_{h}^{\alpha}=\left(\begin{array}{cc} 0 & 0 \\ 0 & \Gamma_{h,B}^{\alpha} \end{array} \right), \end{array} $$

where $G_{A/B}^{r/a}$, *Γ*_*e*,*A*_, and *Γ*_*h*,*B*_ are 3×3 matrices. So

$$\begin{array}{@{}rcl@{}} Tr\left[G^{r}\Gamma_{e}^{\alpha}G^{a}\Gamma_{h}^{\beta}\right]=Tr \left[\left(\begin{array}{cc} {G_{A}^{r}} \Gamma_{e,A}^{\alpha} & 0 \\ 0 & 0 \end{array} \right) \left(\begin{array}{cc} 0 & 0 \\ 0 & {G_{B}^{a}}\Gamma_{h,B}^{\beta} \end{array} \right)\right]=0. \end{array} $$

**Figure 10:**
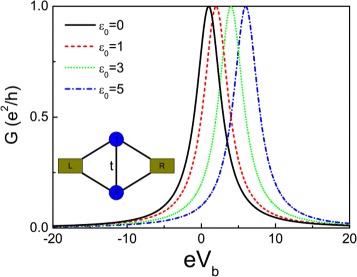
Conductance curves of directly parallel coupled double quantum dot system with various electron levels in quantum dots. The energy levels of the two quantum dots are tuned synchronously *ε*_1_=*ε*_2_=*ε*_0_. The dot-lead coupling *Γ*_*L*1_=*Γ*_*L*2_=*Γ*_*R*1_=*Γ*_*R*2_=2.0. The coupling strength of the two dots *t* is set as the unit of energy.

We further perform the calculation based on the model of two quantum dots connected by Kitaev chain (in the topological nontrivial phase with MF on each end of the chain) and parallel connected to two leads. We find complete suppression of the local AR and CAR for *ε*_1_=*ε*_2_, which is consistent with the conclusion based on the Hamiltonian (1)-(2). Based on Green’s function technique, the conductance of the directly parallel coupled double quantum dot system is calculated (see Figure 10), one can see details discussion in Appendix 3.

## Conclusions

The transport properties through the parallel coupled QD-NW-QD system have been studied, in which a particular attention is paid to the mechanism of the Fano resonance in conductance spectrum of systems with symmetric configuration.

In the case of long nanowire without interaction between MFs (*ε*_*M*_=0), the conductance exhibits Breit-Wigner and Fano resonances with positions and Fano factors controlled by the energy level of the QDs. When *ε*_*M*_ is not equal to zero for short nanowires, there is a nanowire length-dependent Fano resonance. In particular, a clear relationship between the Fano factor and MBS coupling strength *ε*_*M*_ has been revealed, which can be used to measure the length at nanometer scale. Our method of detection of MFs based on Fano resonances in hybrid nanostructures has the advantages of tunability and robust to external perturbation and noise.

## Appendices

### Appendix 1

#### More details of the theoretical formalism

The total Hamiltonian of the QD-NW-QD system is:

(12) $$\begin{array}{@{}rcl@{}} H=H_{\text{system}}+H_{\text{leads}}+H_{T}, \end{array} $$

where:

$$\begin{array}{@{}rcl@{}} H_{\text{system}} &=&\varepsilon_{1}d_{1}^{\dagger}d_{1}+\varepsilon_{2}d_{2}^{\dagger}d_{2}+\frac{i}{2}\epsilon_{M}\eta_{1}\eta_{2}+t_{1}(d_{1}-d_{1}^{\dagger}) \eta_{1}\\&&+it_{2}(d_{2}^{\dagger}+d_{2})\eta_{2}, \\ H_{\text{leads}} &=&\sum\limits_{\alpha k} \varepsilon_{\alpha k}c_{\alpha k}^{\dagger}c_{\alpha k}, \\ H_{T} &=&\sum\limits_{\alpha j} V_{\textit{j}\alpha}d_{j}^{\dagger}c_{\alpha k}+H.c. \end{array} $$

Using the relation *η*_1_=*f*+*f*^*†*^,*η*_2_=*i*(*f*^*†*^−*f*), we transform the Majorana operators to Dirac operators.

Then,

(13) $$\begin{array}{@{}rcl@{}} H_{\text{system}} &=&\varepsilon_{1} d_{1}^{\dagger}d_{1}+\varepsilon_{2}d_{2}^{\dagger}d_{2}+\epsilon_{M}(f^{\dagger}f-\frac{1}{2}) \\  &&+t_{1}(d_{1}-d_{1}^{\dagger})(f+f^{\dagger})-t_{2}(d_{2}^{\dagger}+d_{2})(f^{\dagger}-f). \end{array} $$

In the Nambu representation which is spanned by $\Psi =(d_{1},d_{1}^{\dagger },f,f^{\dagger },d_{2},d_{2}^{\dagger })^{T}$, the Hamiltonian can be written as:

(14) $$\begin{array}{@{}rcl@{}} H_{\text{system}}=\frac{1}{2}\Psi^{\dagger}H_{\text{BdG}}\Psi, \end{array} $$

where:

$$\begin{array}{@{}rcl@{}} H_{\text{BdG}}=\left(\begin{array}{cccccc} \varepsilon_{1} & 0 & -t_{1} & -t_{1} & 0 & 0 \\ 0 & -\varepsilon_{1} & t_{1} & t_{1} & 0 & 0 \\ -t_{1} & t_{1} & \epsilon_{M} & 0 & t_{2} & t_{2} \\ -t_{1} & t_{1} & 0 & -\epsilon_{M} & -t_{2} & -t_{2} \\ 0 & 0 & t_{2} & -t_{2} & \varepsilon_{2} & 0 \\ 0 & 0 & t_{2} & -t_{2} & 0 & -\varepsilon_{2} \end{array} \right). \end{array} $$

The current flowing from the left lead to the central region can be defined from the rate of change of the electron number $N_{L}=\sum _{k}c_{\textit {Lk}}^{\dagger }c_{\textit {Lk}}$ in the left lead. Following Meir and Wingreen [57], the current can be formulated in Nambu space:

$$\begin{array}{@{}rcl@{}} I_{L} &=&-e \langle \dot{N}_{L} \rangle=-i\frac{e}{\hbar} \left\langle\left[H_{T},N_{L}\right]\right\rangle \\ &=&\frac{e}{\hbar}\sum\limits_{n,k}[(V_{1L}^{\ast}\delta_{1n}+V_{2L}^{\ast}\delta_{5n})G_{n,Lk}^{<}\left(t,t\right) -(V_{1L}\delta_{1n}\\&&+V_{2L}\delta_{5n})G_{Lk,n}^{<}\left(t,t\right)], \end{array} $$

where:

$$\begin{array}{@{}rcl@{}} G_{n,Lk}^{<}(t,t^{^{\prime}}) =i \langle c_{Lk}^{\dagger}\left(t^{\prime}\right)\Psi_{n}\left(t\right) \rangle, && G_{Lk,n}^{<}(t,t^{^{\prime}}) =i \langle \Psi_{n}^{\dagger}\left(t^{\prime}\right)c_{Lk}\left(t\right) \rangle. \end{array} $$

The equations of motion for $G_{n,Lk}^{<}$ and $ G_{Lk,n}^{<}$ along with the Langreth analytic continuation yield the following equations:

(15) $$\begin{array}{@{}rcl@{}} G_{n,Lk}^{<}\left(t,t\right)&=&\int dt_{1}\sum\limits_{m}(V_{1L}\delta_{1m}+V_{2L}\delta_{5m})\\&&\times\left[G_{nm}^{r}\left(t,t_{1}\right) g_{Lke}^{<}(t_{1},t)\right.  \\ & &\left.+G_{nm}^{<}\left(t,t_{1}\right) g_{Lke}^{a}(t_{1},t)\right], \end{array} $$

(16) $$\begin{array}{@{}rcl@{}} G_{Lk,n}^{<}\left(t,t\right)&=&\int dt_{1}\sum\limits_{m}(V_{1L}^{\ast}\delta_{1m}+V_{2L}^{\ast}\delta_{5m})\\ &&\times\left[g_{Lke}^{r}\left(t,t_{1}\right) G_{mn}^{<}(t_{1},t)\right.  \\ & &\left.+g_{Lke}^{<}\left(t,t_{1}\right)G_{mn}^{a}(t_{1},t)\right.], \end{array} $$

in which $g_{\textit {Lke}}^{r/a}$ and $g_{\textit {Lke}}^{</>}$ are the unperturbed retarded/advanced and lesser/greater Green’s functions for electron of the left lead, respectively. Substituting Equations (15) and (16) into the current formula, one can obtain:

(17) $$ {\fontsize{9.5}{6} \begin{aligned} I_{L}\left(t\right)=&\frac{e}{h}\int dt_{1}Tr[G^{r}\left(t,t_{1}\right)\Sigma_{Le}^{<}(t_{1},t)+G^{<}\left(t,t_{1}\right) \Sigma_{Le}^{a}(t_{1},t) \\ &-\Sigma_{Le}^{r}\left(t,t_{1}\right)G^{<}(t_{1},t)-\Sigma_{Le}^{<}\left(t,t_{1}\right) G^{a}(t_{1},t)], \end{aligned}}  $$

where the trace is over the Nambu space. *G*^*r*/<^ is the retarded and/or lesser Green’s function, which can be derived from the analytical continuation of the contour-ordered Green’s function *G*(*t*,*t*^′^)=−*i*〈*TΨ*(*t*)*Ψ*^*†*^(*t*^′^)〉. Performing Fourier transformation, the current reads:

(18) $$\begin{array}{@{}rcl@{}} I_{L}&=&\frac{e}{h}\int d\omega Tr\left[G^{r}\left(\omega\right)\Sigma_{Le}^{<}(\omega)+G^{<}\left(\omega\right) \Sigma_{Le}^{a}(\omega)\right.  \\ &&\left.-\Sigma_{Le}^{r}\left(\omega\right)G^{<}(\omega)-\Sigma_{Le}^{<}\left(\omega\right)G^{a}(\omega)\right]. \end{array} $$

The retarded Green’s function of the system is formally given by the Dyson equation:

(19) $$\begin{array}{@{}rcl@{}} G^{r}(\omega)=(\omega-H_{\text{BdG}}-\Sigma^{r})^{-1}, \end{array} $$

where the retarded self-energy *Σ*^*r*^ is due to the coupling to the leads, i.e., $\Sigma ^{r}=\sum _{\alpha }(\Sigma _{\alpha e}^{r}+ \Sigma _{\alpha h}^{r})$. In the wide-band limit, they are given, respectively, by $\Sigma _{\alpha e}^{r}=-\frac {i}{2}\Gamma _{e}^{\alpha }$, $\Sigma _{\alpha h}^{r}=-\frac {i}{2}\Gamma _{h}^{\alpha }$. Here, $\Gamma _{e}^{\alpha } (\Gamma _{h}^{\alpha })$ are 6 ×6 matrices describing the coupling of particle (hole) to *α* lead:

$$\begin{array}{@{}rcl@{}} \Gamma_{e,mn}^{\alpha} &=&\Gamma_{\alpha 1}\delta_{1m}\delta_{1n}+\Gamma_{\alpha 2}\delta_{5m}\delta_{5n}+\sqrt{\Gamma_{\alpha 1}\Gamma_{\alpha 2}}(\delta_{1m}\delta_{5n}\\&&+\delta_{5m}\delta_{1n}) \\ \Gamma_{h,mn}^{\alpha} &=&\Gamma_{\alpha 1}\delta_{2m}\delta_{2n}+\Gamma_{\alpha 2}\delta_{6m}\delta_{6n}+\sqrt{\Gamma_{\alpha 1}\Gamma_{\alpha 2}}(\delta_{2m}\delta_{6n}\\&&+\delta_{6m}\delta_{2n}), \end{array} $$

where *Γ*_*αj*_=2*π*|*V*_*jα*_|^2^*ρ*_*α*_ is the dot-lead coupling and *ρ*_*α*_ is the density of states of the *α* lead. We use the relation:

(20) $$\begin{array}{@{}rcl@{}} G^{r}-G^{a}=G^{>}-G^{<} \end{array} $$

together with the Keldysh equation:

(21) $$\begin{array}{@{}rcl@{}} G^{</>}=G^{r}\Sigma^{</>}G^{a}, \end{array} $$

where the advanced Green’s function *G*^*a*^=(*G*^*r*^)^*†*^, the lesser/greater self-energy $\Sigma ^{</>}=\sum _{\alpha }(\Sigma _{\alpha e}^{</>}+\Sigma _{\alpha h}^{</>})$, and $\Sigma _{\alpha e/h}^{</>}=i\Gamma _{e/h}^{\alpha }(f_{e/h}^{\alpha }-\frac {1}{2}\pm \frac {1}{2})$. Here, $f_{e}^{\alpha }$ and $f_{h}^{\alpha }$ are the Fermi distribution functions of the electron and hole in *α* lead, respectively, i.e.,

$$\begin{array}{@{}rcl@{}} f_{e}^{\alpha}\left(\omega\right) =(1+e^{\frac{\omega -\mu_{\alpha}}{k_{B}T}})^{-1}, \qquad & f_{h}^{\alpha}\left(\omega\right) =(1+e^{\frac{\omega +\mu_{\alpha}}{k_{B}T}})^{-1}. \end{array} $$

Substituting Equations (20) and (21) into Equation (17), we obtain:

(22) $$ {\fontsize{9.5}{6}\begin{aligned} I_{L} =&\,\frac{e}{h}\int d\omega Tr\left[G^{r}\Sigma^{>}G^{a}\Sigma_{Le}^{<}-G^{r}\Sigma^{<}G^{a}\Sigma_{Le}^{>}\right] \\ =&\,\frac{e}{h}\int d\omega \{Tr\left[G^{r}{\Gamma_{h}^{L}}G^{a}{\Gamma_{e}^{L}}\right]\left({f_{e}^{L}}-{f_{h}^{L}}\right)+Tr\left[G^{r}{\Gamma_{e}^{R}} G^{a}{\Gamma_{e}^{L}}\right] \\ &\left({f_{e}^{L}}-{f_{e}^{R}}\right)+Tr\left[G^{r}{\Gamma_{h}^{R}}G^{a}{\Gamma_{e}^{L}}\right]\left({f_{e}^{L}}-{f_{h}^{R}}\right)\Big\}. \end{aligned}}  $$

After solving the Dyson and Keldysh equations, one is ready to obtain the current from Equation (22).

### Appendix 2

#### The molecular states and the density of states of case I without interaction between MFs

We consider the symmetric configuration *ε*_1_=*ε*_2_=*ε*_0_, *t*_1_=*t*_2_=*t*, and *ε*_*M*_=0. By diagonalizing the system Hamiltonian in the Nambu representation, the solutions to the Bogoliubov-de Gennes equations *H*_BdG_*ψ*_*i*_=*E*_*i*_*ψ*_*i*_ are obtained as:

$$\begin{array}{@{}rcl@{}} \psi_{1} &=&\frac{1}{\sqrt{2}}\left(\begin{array}{cccccc} \frac{2t}{\Delta} & \frac{2t}{\Delta} & \frac{\varepsilon_{0}}{\Delta} & \frac{\varepsilon_{0}}{\Delta} & 0 & 0 \end{array} \right), E_{1}=0, \\ \psi_{2} &=&\frac{i}{\sqrt{2}}\left(\begin{array}{cccccc} 0 & 0 & \frac{\varepsilon_{0}}{\Delta} & -\frac{\varepsilon_{0}}{\Delta} & -\frac{2t}{\Delta} & \frac{2t}{\Delta} \end{array} \right), E_{2}=0, \\ \psi_{3} &=&\left(\begin{array}{cccccc} \frac{\varepsilon_{0}+\Delta}{\sqrt{8}\Delta} & \frac{\varepsilon_{0}-\Delta}{\sqrt{8}\Delta} & \frac{-4t}{\sqrt{8}\Delta} & 0 & \frac{-\varepsilon_{0}-\Delta}{\sqrt{8}\Delta} & \frac{\varepsilon_{0}-\Delta}{\sqrt{8}\Delta} \end{array} \right), E_{3}=\Delta, \\ \psi_{4} &=&\left(\begin{array}{cccccc} \frac{\varepsilon_{0}-\Delta}{\sqrt{8}\Delta} & \frac{\varepsilon_{0}+\Delta}{\sqrt{8}\Delta} & 0 & \frac{-4t}{\sqrt{8}\Delta} &\frac{\varepsilon_{0}-\Delta}{\sqrt{8}\Delta} & \frac{-\varepsilon_{0}-\Delta}{\sqrt{8}\Delta} \end{array} \right), E_{4}=-\Delta, \\ \psi_{5} &=&\left(\begin{array}{cccccc} \frac{\varepsilon_{0}+\Delta}{\sqrt{8}\Delta} & \frac{\varepsilon_{0}-\Delta}{\sqrt{8}\Delta} & 0 & \frac{-4t}{\sqrt{8}\Delta} & \frac{\varepsilon_{0}+\Delta}{\sqrt{8}\Delta} & \frac{\Delta-\varepsilon_{0}}{\sqrt{8}\Delta} \end{array} \right), E_{5}=\Delta, \\ \psi_{6} &=&\left(\begin{array}{cccccc} \frac{\varepsilon_{0}-\Delta}{\sqrt{8}\Delta} & \frac{\varepsilon_{0}+\Delta}{\sqrt{8}\Delta} & \frac{-4t}{\sqrt{8}\Delta} & 0 & \frac{\Delta-\varepsilon_{0}}{\sqrt{8}\Delta} & \frac{\varepsilon_{0}+\Delta}{\sqrt{8}\Delta} \end{array} \right), E_{6}=-\Delta, \end{array} $$

where $\Delta =\sqrt {{\varepsilon _{0}^{2}}+4t^{2}}$. For the two zero-energy states, one can find $\gamma _{1}=\psi _{1}=\frac {1}{\sqrt {2}}\left (\frac {2t}{\Delta }d_{1}+\frac {2t}{\Delta } d_{1}^{\dagger }+\frac {\varepsilon _{0}}{\Delta }f+\frac {\varepsilon _{0}}{ \Delta }f^{\dagger }\right)$ and $\gamma _{2}=\psi _{2}=\frac {i}{\sqrt {2}}\left (\frac {\varepsilon _{0}}{\Delta }f^{\dagger }-\frac {\varepsilon _{0}}{\Delta }f+\frac {2t}{\Delta }d_{2}-\frac {2t}{\Delta }d_{2}^{\dagger }\right)$ which satisfy $\gamma _{1,2}=\gamma _{1,2}^{\dagger }$. They are Majorana bound states. If *ε*_0_=0, the two MBSs are spatially isolated since each zero-energy mode is completely localized on one of the dots.

The Majorana operators can be transformed into Dirac operators,

$$\begin{array}{@{}rcl@{}} \tilde{c}_{1}^{\dagger} =\frac{1}{\sqrt{2}}\left(\gamma_{1}-i\gamma_{2}\right), \qquad&& \tilde{c}_{1} =\frac{1}{\sqrt{2}}\left(\gamma_{1}+i\gamma_{2}\right). \end{array} $$

Then, one can make the following transformation of the Dirac operators:

$$\begin{array}{@{}rcl@{}} \tilde{c}_{1} &=&\frac{t}{\Delta}d_{1}+\frac{t}{\Delta}d_{1}^{\dagger}+\frac{\varepsilon_{0}}{\Delta}f^{\dagger}+\frac{t}{ \Delta}d_{2}-\frac{t}{\Delta}d_{2}^{\dagger}, \\ \tilde{c}_{1}^{\dagger} &=&\frac{t}{\Delta}d_{1}+\frac{t}{\Delta}d_{1}^{\dagger}+ \frac{\varepsilon_{0}}{\Delta}f-\frac{t}{\Delta}d_{2}+\frac{t}{\Delta}d_{2}^{\dagger}, \\ \tilde{c}_{2} &=&\frac{\varepsilon_{0}-\Delta}{\sqrt{8}\Delta}d_{1}+\frac{\varepsilon_{0}+\Delta}{\sqrt{8}\Delta} d_{1}^{\dagger}-\frac{\sqrt{2}t}{\Delta}f^{\dagger}+\frac{\varepsilon_{0}-\Delta}{\sqrt{8}\Delta}d_{2}\\ &&-\frac{\varepsilon_{0}+\Delta}{\sqrt{8}\Delta}d_{2}^{\dagger}, \\ \tilde{c}_{2}^{\dagger} &=&\frac{\varepsilon_{0}+\Delta}{\sqrt{8}\Delta}d_{1}+\frac{\varepsilon_{0}-\Delta}{\sqrt{8}\Delta} d_{1}^{\dagger}-\frac{\sqrt{2}t}{\Delta}f-\frac{\varepsilon_{0}+\Delta}{\sqrt{8}\Delta}d_{2}\\ &&+\frac{\varepsilon_{0}-\Delta}{\sqrt{8}\Delta}d_{2}^{\dagger}, \\ \tilde{c}_{3} &=&\frac{\varepsilon_{0}+\Delta}{\sqrt{8}\Delta}d_{1}+\frac{\varepsilon_{0}-\Delta}{\sqrt{8}\Delta} d_{1}^{\dagger}-\frac{\sqrt{2}t}{\Delta}f^{\dagger}+\frac{\varepsilon_{0}+\Delta}{\sqrt{8}\Delta}d_{2}\\ &&+\frac{\Delta-\varepsilon_{0}}{\sqrt{8}\Delta}d_{2}^{\dagger}, \\ \tilde{c}_{3}^{\dagger} &=&\frac{\varepsilon_{0}-\Delta}{\sqrt{8}\Delta}d_{1}+\frac{\varepsilon_{0}+\Delta}{\sqrt{8}\Delta} d_{1}^{\dagger}-\frac{\sqrt{2}t}{\Delta}f+\frac{\Delta-\varepsilon_{0}}{\sqrt{8}\Delta}d_{2}\\ &&+\frac{\varepsilon_{0}+\Delta}{\sqrt{8}\Delta}d_{2}^{\dagger}. \end{array} $$

Thus, the Hamiltonian of the middle system becomes diagonal with the form

$$\begin{array}{@{}rcl@{}} H_{\text{system}}=\tilde{\varepsilon}_{1}\tilde{c}_{1}^{\dagger}\tilde{c}_{1}+\tilde{\varepsilon}_{2}\tilde{c}_{2}^{\dagger}\tilde{c}_{2}+\tilde{ \varepsilon}_{3}\tilde{c}_{3}^{\dagger}\tilde{c}_{3}, \end{array} $$

with $\tilde {\varepsilon }_{1}=0$, $\tilde {\varepsilon }_{2}=-\Delta $, $\tilde {\varepsilon }_{3}=\Delta $. In the above molecular state representation with symmetric coupling of the middle system to the leads, i.e., $V_{1\alpha }=V_{1\alpha }^{\ast }=V_{2\alpha }=V_{2\alpha }^{\ast }=V_{\alpha }$, the tunneling Hamiltonian between the leads and the molecular states is written as:

$$\begin{array}{@{}rcl@{}} \tilde{H}_{T}=\sum_{\alpha k}V_{\alpha}\left(\frac{2t}{\Delta}\tilde{c}_{1}^{\dagger}+\frac{\varepsilon_{0}-\Delta}{\sqrt{2}\Delta}\tilde{c} _{2}^{\dagger}+\frac{\varepsilon_{0}+\Delta}{\sqrt{2}\Delta}\tilde{c}_{3}^{\dagger}\right)c_{\alpha k}+H.c. \end{array} $$

Notice that only three molecular states coupled to the leads because of the symmetry of the system. The broadening of the molecular states due to their coupling to leads can be given by the DOS of each state. With the help of the equation of motion approach, one can obtain:

$$\begin{array}{@{}rcl@{}} G_{11}^{r}\left(\omega\right) &=&\frac{1}{\omega-\tilde{\varepsilon}_{1}+i\Gamma_{1}}, \\ G_{22}^{r}\left(\omega\right) &=&\frac{1}{\omega-\tilde{\varepsilon}_{2}+i\Gamma_{2}}, \\ G_{33}^{r}\left(\omega\right) &=&\frac{1}{\omega-\tilde{\varepsilon}_{3}+i\Gamma_{3}}, \end{array} $$

where the widths of the three molecular states read:

$$\begin{array}{@{}rcl@{}} \Gamma_{1} &=&\frac{2t^{2}}{\Delta^{2}}(\Gamma_{L}+\Gamma_{R}), \\ \Gamma_{2} &=&\frac{\left(\varepsilon_{0}-\Delta\right)^{2}}{4\Delta^{2}}(\Gamma_{L}+\Gamma_{R}), \\ \Gamma_{3} &=&\frac{\left(\varepsilon_{0}+\Delta\right)^{2}}{4\Delta^{2}}(\Gamma_{L}+\Gamma_{R}), \end{array} $$

and *Γ*_*α*_=2*π*|*V*_*α*_|^2^*ρ*_*α*_. The local density of states is defined as the imaginary part of the retarded Green’s function as:

$$\begin{array}{@{}rcl@{}} \rho_{1} &=&-\frac{1}{\pi}\text{Im}G_{11}^{r}\left(\omega\right)=\frac{1 }{\pi}\frac{\Gamma_{1}}{\left(\omega-\tilde{\varepsilon}_{1}\right)^{2}+\left(\Gamma_{1}\right)^{2}}, \\ \rho_{2} &=&-\frac{1}{\pi}\text{Im}G_{22}^{r}\left(\omega\right)=\frac{1 }{\pi}\frac{\Gamma_{2}}{\left(\omega-\tilde{\varepsilon}_{2}\right)^{2}+\left(\Gamma_{2}\right)^{2}}, \\ \rho_{3} &=&-\frac{1}{\pi}\text{Im}G_{33}^{r}\left(\omega\right)=\frac{1 }{\pi}\frac{\Gamma_{3}}{\left(\omega-\tilde{\varepsilon}_{3}\right)^{2}+\left(\Gamma_{3}\right)^{2}}. \end{array} $$

Based on the equation of motion method, the analytical form of differential conductance reads:

$${\fontsize{8.8}{6}\begin{aligned} G=\frac{d I}{d V_{b}}=\frac{e^{2}}{h}\frac{4\Gamma^{2}[2t^{2}-\omega(\varepsilon_{0}+\omega)]^{2}} {4\Gamma^{2}[2t^{2}-\omega(\varepsilon_{0}+\omega)]^{2}+\omega^{2}(\Delta^{2}-\omega^{2})^{2}} \Big|_{\omega=eV_{b}}. \end{aligned}} $$

### Appendix 3

#### The conductance of the directly parallel coupled double quantum dots

Here, the electron energy levels of the two quantum dots are set aligned with each other by the gate voltage. In the symmetric configuration, one sees the symmetric Breit-Wigner line shapes and the conductance curve shifts trivially with the energy level of the quantum dots. The coupling strength of the two dots *t* is set as the unit of energy.

### Appendix 4

#### The molecular states and the density of states of case II with interaction between MFs

In this case, we assume *ε*_1_=*ε*_2_=0, and *t*_1_=*t*_2_=*t*. By diagonalizing the system Hamiltonian in the Nambu representation, the solutions to the Bogoliubov-de Gennes equations *H*_*BdG*_*ψ*_*i*_=*E*_*i*_*ψ*_*i*_ are:

$$\begin{aligned} \psi_{1} &=\frac{1}{\sqrt{2}}\left(\begin{array}{cccccc} 1 & 1 & 0 & 0 & 0 & 0 \end{array} \right), E_{1}=0, \\ \psi_{2} &=\frac{i}{\sqrt{2}}\left(\begin{array}{cccccc} 0 & 0 & 0 & 0 & -1 & 1 \end{array} \right), E_{2}=0, \\ \psi_{3} &=\frac{1}{2\sqrt{2}}\left(\frac{\kappa+\epsilon_{M}}{ \kappa}\right)^{\frac{1}{2}}\left(\begin{array}{cccccc} -1 & 1 & \frac{\epsilon_{M}-\kappa }{2t} & 0 & 1 & 1 \end{array} \right),\\ E_{3}&=-\frac{\epsilon_{M}-\kappa}{2}, \\ \psi_{4} &=\frac{1}{2\sqrt{2}}\left(\frac{\kappa+\epsilon_{M}}{ \kappa}\right)^{\frac{1}{2}}\left(\begin{array}{cccccc} 1 & -1 & 0 & \frac{\epsilon_{M}-\kappa}{2t} & 1 & 1 \end{array} \right),\\ E_{4}&=\frac{\epsilon_{M}-\kappa }{2}, \\ \psi_{5} &=\frac{1}{2\sqrt{2}}\left(\frac{\kappa-\varepsilon_{M}}{ \kappa}\right)^{\frac{1}{2}}\left(\begin{array}{cccccc} -1 & 1 & \frac{\epsilon_{M}+\kappa}{2t} & 0 & 1 & 1 \end{array} \right),\\ E_{5}&=\frac{\epsilon_{M}+\kappa}{2}, \\ \psi_{6} &=\frac{1}{2\sqrt{2}}\left(\frac{\kappa-\varepsilon_{M}}{ \kappa}\right)^{\frac{1}{2}}\left(\begin{array}{cccccc} 1 & -1 & 0 & \frac{\epsilon_{M}+\kappa}{2t} & 1 & 1 \end{array} \right),\\ E_{6}&=-\frac{\epsilon_{M}+\kappa}{2}, \end{aligned} $$

where $\kappa =\sqrt {{\epsilon _{M}^{2}}+16t^{2}}$. One can find $\gamma _{1}=\psi _{1}=\frac {1}{\sqrt {2}}\left (d_{1}+d_{1}^{\dagger }\right)$ and $\gamma _{2}=\psi _{2}=\frac {i}{\sqrt {2}}\left (d_{2}^{\dagger }-d_{2}\right)$ satisfy $\gamma _{1,2}=\gamma _{1,2}^{\dagger }$. They are Majorana bound states which are always spatially isolated. The Majorana operators can be transformed into Dirac operators:

$$\begin{array}{@{}rcl@{}} \tilde{c}_{1}^{\dagger} =\frac{1}{\sqrt{2}}\left(\gamma_{1}-i\gamma_{2}\right), \qquad&& \tilde{c}_{1} =\frac{1}{\sqrt{2}}\left(\gamma_{1}+i\gamma_{2}\right). \end{array} $$

Then, one can make the following transformation of the Dirac operators:

$${\fontsize{8.8}{6}\begin{aligned} \tilde{c}_{1} &=\frac{1}{2}\left(d_{1}+d_{1}^{\dagger}+d_{2}-d_{2}^{\dagger}\right), \\ \tilde{c}_{1}^{\dagger} &=\frac{1}{2}\left(d_{1}+d_{1}^{\dagger}-d_{2}+d_{2}^{\dagger}\right), \\ \tilde{c}_{2} &=\frac{1}{2\sqrt{2}}\left(\frac{\kappa+\epsilon_{M}}{\kappa}\right)^{\frac{1}{2}}\left[d_{1}-d_{1}^{\dagger}+\frac{ \epsilon_{M}-\kappa}{2t}f^{\dagger}+d_{2}+d_{2}^{\dagger}\right], \\ \tilde{c}_{2}^{\dagger} &=\frac{1}{2\sqrt{2}}\left(\frac{\kappa+\epsilon_{M}}{\kappa}\right)^{\frac{1}{2}}\left[-d_{1}+d_{1}^{ \dagger}+\frac{\epsilon_{M}-\kappa}{2t}f+d_{2}+d_{2}^{\dagger}\right],\\ \tilde{c}_{3} &=\frac{1}{2\sqrt{2}}\left(\frac{\kappa-\epsilon_{M}}{\kappa}\right)^{\frac{1}{2}}\left[ d_{1}-d_{1}^{\dagger }+\frac{\epsilon_{M}+\kappa}{2t}f^{\dagger}+d_{2}+d_{2}^{\dagger}\right],\\ \tilde{c}_{3}^{\dagger} &=\frac{1}{2\sqrt{2}}\left(\frac{\kappa-\epsilon_{M}}{ \kappa}\right)^{\frac{1}{2}}\left[-d_{1}+d_{1}^{\dagger}+\frac{\epsilon_{M}+\kappa}{2t}f+d_{2}+d_{2}^{\dagger}\right]. \end{aligned}} $$

Thus, the Hamiltonian of the middle system becomes diagonal with the form:

$$\begin{array}{@{}rcl@{}} H_{\text{system}}=\tilde{\varepsilon}_{1}\tilde{c}_{1}^{\dagger}\tilde{c}_{1}+ \tilde{\varepsilon}_{2}\tilde{c}_{2}^{\dagger}\tilde{c}_{2}+\tilde{\varepsilon}_{3}\tilde{c}_{3}^{\dagger}\tilde{c}_{3}, \end{array} $$

with $\tilde {\varepsilon }_{1}=0$, $\tilde {\varepsilon }_{2}=\frac {\kappa -\epsilon _{M}}{2}$, and $\tilde {\varepsilon }_{3}=-\frac {\kappa +\epsilon _{M}}{2}$. In the above molecular state representation with symmetric coupling of the middle system to the leads, i.e., $V_{1\alpha }=V_{1\alpha }^{\ast }=V_{2\alpha }=V_{2\alpha }^{\ast }=V_{\alpha }$, the tunneling Hamiltonian between the leads and the molecular states is written as:

$$\begin{array}{@{}rcl@{}} \tilde{H}_{T}\!&=&\!\sum_{\alpha k }V_{\alpha}\left[\tilde{c}_{1}^{\dagger}+\left(\frac{\kappa+\epsilon_{M}}{2\kappa}\right)^{\frac{1}{2}}\tilde{c}_{2}^{\dagger} +\left(\frac{\kappa-\epsilon_{M}}{2\kappa}\right)^{\frac{1}{2}}\tilde{c}_{3}^{\dagger}\right] c_{\alpha k}\\&&\,+H.c. \end{array} $$

Notice that because of the symmetry of the system, only three molecular states couple to the leads. The broadening of the molecular states due to their coupling to leads can be given by the DOS of each state. With the help of the equation of motion approach, one can obtain:

$$\begin{array}{@{}rcl@{}} G_{11}^{r}\left(\omega\right) &=&\frac{1}{\omega-\tilde{\varepsilon}_{1}+i\Gamma_{1}}, \\ G_{22}^{r}\left(\omega\right) &=&\frac{1}{\omega-\tilde{\varepsilon}_{2}+i\Gamma_{2}}, \\ G_{33}^{r}\left(\omega\right) &=&\frac{1}{\omega-\tilde{\varepsilon}_{3}+i\Gamma_{3}}, \end{array} $$

where the widths of the three molecular states read:

$$\begin{array}{@{}rcl@{}} \Gamma_{1} &=&\frac{1}{2}(\Gamma_{L}+\Gamma_{R}), \\ \Gamma_{2} &=&\frac{\kappa+\epsilon_{M}}{4\kappa}(\Gamma_{L}+\Gamma_{R}), \\ \Gamma_{3} &=&\frac{\kappa-\epsilon_{M}}{4\kappa}(\Gamma_{L}+\Gamma_{R}). \end{array} $$

The local density of states is defined as the imaginary part of the retarded Green’s function as:

$$\begin{array}{@{}rcl@{}} \rho_{1} &=& -\frac{1}{\pi}\text{Im}G_{11}^{r}\left(\omega\right)=\frac{1}{\pi} \frac{\Gamma_{1}}{\left(\omega-\tilde{\varepsilon}_{1}\right)^{2}+\left(\Gamma_{1}\right)^{2}} \\ \rho_{2} &=&-\frac{1}{\pi}\text{Im}G_{22}^{r}\left(\omega\right)=\frac{1 }{\pi}\frac{\Gamma_{2}}{\left(\omega-\tilde{\varepsilon}_{2}\right)^{2}+\left(\Gamma_{2}\right)^{2}} \\ \rho_{3} &=&-\frac{1}{\pi}\text{Im}G_{33}^{r}\left(\omega\right)=\frac{1 }{\pi}\frac{\Gamma_{3}}{\left(\omega-\tilde{\varepsilon}_{3}\right)^{2}+\left(\Gamma_{3}\right)^{2}}. \end{array} $$

Based on the equation of motion method, the analytical form of differential conductance reads:

$${\fontsize{9}{6}\begin{aligned} G&=\frac{d I}{d V_{b}}\\&=\frac{e^{2}}{h}\frac{\Gamma^{2}[4t^{2}-2\epsilon_{M}\omega-\omega^{2}]^{2}} {\Gamma^{2}[4t^{2}-2\epsilon_{M}\omega-\omega^{2}]^{2}+\omega^{2}(4t^{2}-\epsilon_{M}\omega-\omega^{2})^{2}} \Big|_{\omega=eV_{b}}. \end{aligned}} $$
